# The key role of local and global farmer networks in the development of conservation agriculture in California

**DOI:** 10.1002/jeq2.70039

**Published:** 2025-05-22

**Authors:** J. P. Mitchell, L. E. Jackson, D. C. Reicosky, A. Kassam, A. Shrestha, R. Harben, E. M. Miyao, K. M. Scow, G. Sposito, D. Beck, T. Friedrich, A. S. Mitchell, R. Schmidt, S. Park, B. Park, P. Foster, P. Muller, A. Brait, T. Willey, M. Bottens, C. Crum, D. Giacomazzi, T. Barcellos, M. V. Crowell, R. Roy, H. Ferris, J. L. Chiartas, E. Brennan, A. Gaudin, John Diener, Justin Diener, L. Asgill, E. A. Kueneman, J. Fisher, M. Bartz, R. A. Peiretti, R. Derpsch, J. Landers, B. J. Aegerter, M. Leinfelder‐Miles, S. E. Light, J. McPhee, R. B. Ferraz Branco

**Affiliations:** ^1^ Department of Plant Sciences University of California Davis California USA; ^2^ Department of Land, Air and Water Resources University of California Davis California USA; ^3^ Retired, USDA ARS North Central Soil Conservation Research Laboratory Morris Minnesota USA; ^4^ University of Reading Reading United Kingdom; ^5^ Department of Plant Sciences California State University Fresno California USA; ^6^ Retired, USDA Natural Resources Conservation Service Arroyo Grande California USA; ^7^ Retired, University of California Cooperative Extension Woodland California USA; ^8^ Department of Environmental Science, Policy, and Management University of California Berkeley California USA; ^9^ Dakota Lakes Research Farm Pierre South Dakota USA; ^10^ Ex‐FAO Senior Expert and Representative (retired) Sillerup Germany; ^11^ Department of Radiology, Health Center University of California, Davis Sacramento California USA; ^12^ Working Lands Innovation Center University of California, Davis Institute of the Environment Davis California USA; ^13^ Park Farming Meridian California USA; ^14^ Pinnacle Organic Produce Hollister California USA; ^15^ Full Belly Farms Guinda California USA; ^16^ T & D Willey Farms Madera California USA; ^17^ California Ag Solutions Madera California USA; ^18^ Agrotechnovation Clovis California USA; ^19^ Giacomazzi Dairy Hanford California USA; ^20^ T‐Bar Dairy Tipton California USA; ^21^ Bar‐Vee Dairy Turlock California USA; ^22^ USDA Natural Resources Conservation Service Fresno California USA; ^23^ Department of Entomology and Nematology University of California Davis California USA; ^24^ REGENScore Davis California USA; ^25^ USDA ARS Salinas California USA; ^26^ Red Rock Ranch Five Points California USA; ^27^ Ecoinnovation, Inc. Petaluma California USA; ^28^ Food and Agriculture Organization of the United Nations West Sacramento California USA; ^29^ Program in Agricultural and Natural Ecosystems at the Federal University of Santa Catarina and Brazilian No‐tillage Systems Farmers’ Federation, Brazil and Center for Functional Ecology, Department of Life Sciences, University of Coimbra Coimbra Portugal; ^30^ Global Farmer Network Foundation Rosario Argentina; ^31^ Independent Consultant Siegen Germany; ^32^ Brazilian No‐Tillage System Farmers’ Federation Brasilia Brazil; ^33^ University of California Cooperative Extension Stockton California USA; ^34^ University of California Cooperative Extension Yuba City California USA; ^35^ Tasmanian Institute of Agricultural Research Burnie Tasmania Australia; ^36^ Instituto Agronômico de Campinas, Centro de Horticultura Campinas Brazil

## Abstract

This article chronicles the history of California's Conservation Agriculture Systems Innovation (CASI) Center and how it has increased agricultural sustainability in the San Joaquin Valley, a major production area for the United States, by using agroecological practices to reduce soil erosion and conserve soil moisture, champion systems thinking, and create networks of farmers, advisors, and researchers. Early conservation agriculture systems in the United States and other continents have informed CASI since its inception in 1998, with an emphasis on reducing soil disturbance for better soil structure and biological activity, retaining biomass on the soil to support soil life, and diversifying crops to enhance biodiversity. CASI includes >2200 farmers, private sector, university, public agency, and environmental group partners. With timelines of its core research and extension education programs, practice adoption trends, and resource quality impacts, CASI's specific accomplishments are described and compared with the dominant tillage‐intensive conventional systems of the past 90 years for crops such as corn, small grains, tomatoes, cotton, dry beans, and melons. An associated 25‐year research station trial has shown that no‐tillage and cover crop practices maintain productivity, increase soil quality (e.g., soil carbon and nitrogen, aggregation, and infiltration), greatly reduce dust that is detrimental to human health, and decrease annual production costs by $50–$75 per acre. CASI tracked a 40‐fold increase in the use of strip‐tillage in dairy silage production during the early 2000s and average annual increases in cover crop seed sales of about 25% in recent years. Outreach, extension, and farmer and industry education programs of CASI include documentary films on YouTube, blogs, workshops, and on‐farm demonstrations. Interactions with other groups and networks are described along with their support for CASI's momentum‐building strategies for impacts. Conservation agriculture is increasing in Central California and continued policy support will enable farmers and institutions to work together to accelerate even greater adoption in the future.

## Core Ideas

- California's Conservation Agriculture Systems Innovation (CASI) Center has fostered farmer‐led innovation for 25 years.
- Many education methods and >2000 participants (farmers, advisors, and researchers) are involved.
- Cover crops and no‐till increased soil quality and maintained crop productivity in a long‐term research trial.
- Conservation agriculture can potentially help California agriculture to remain viable under future water shortages.
- A group of >100 farmers and affiliates is exploring ways to develop organic conservation agriculture for California.

**Abbreviations**
CA: conservation agriculture
CASI: Conservation Agriculture Systems Innovation
CMP: Conservation Management Practice
CT: conservation tillage
NNTF: National No‐till Farmer
NRCS: Natural Resources Conservation Service
NTOP: No‐till on the Plains
PM: particulate matter
PNDSA: Pacific Northwest Direct Seed Association
SJV: San Joaquin Valley

## 1 INTRODUCTION

Based on the need to farm using ecologically sound practices, by the start of the 2000s, major advances had been made in several parts of the world to develop agricultural management systems that in 1997 were first known as “conservation agriculture” (CA) (Duiker, 2021). The core agroecological principles that underlie these systems involve (1) avoiding or minimizing mechanical soil disturbance to maintain soil structure and biology, (2) retaining biomass on the soil to protect and feed soil life and soil quality, and (3) diversifying crop species to enhance biodiversity (Brown, 2018; Mitchell et al., 2024; USDA NRCS, 2012). Later, these goals were included as part of the US federal government's “Unlock the Secrets in the Soil” public awareness campaign to educate people about the benefits of “soil health management” (USDA NRCS, 2012), and more recently as “regenerative agriculture” (Beck, 2014; Newton et al., 2020).

The rise of alternative food production system paradigms, in particular CA, in response to the degrading tillage‐based industrial “Green Revolution” agriculture systems that dominated the mid‐20th century (Kassam & Kassam, 2021), was motivated by a variety of region‐specific technological, environmental, economic, and social evolution factors. Early days of CA in the United States are generally sourced to the 1940s and 1950s following the great “Dust Bowl” era in the 1930s and publication of Plowman's Folly by Edward Faulkner in 1943, largely “in response to the devastation caused by intensive tillage with the moldboard plow” (Duiker, 2021). Then, in Latin America, starting in southern Brazil and eventually in neighboring Argentina, Uruguay, and Paraguay, at the end of the 1960s, where conventional management caused soil degradation and compaction of soil exposed to rain, thereby reducing capacity for water infiltration and leading to large losses of soil to erosion, CA also spread widely. This was the common denominator that spurred farmers from Southern Brazil to travel to the United States to learn no‐tillage techniques developed in the early 1960s, and to create farmer cooperatives, conservation organizations, and alliances of partners to network and adopt no‐till systems that today are used on the majority of annual cropland there (Junior et al., 2012; Kassam et al., 2020). Maize, soybean, cotton, upland rice, and *Phaseolus* beans, along with a range of cover crops are common in South American crop rotations. Improved pasture lands are also commonly rotated with row crops to further enhance soil biodiversity.

Early no‐till innovators and adoption leaders in the United States include Harry Young, Jr., Shirley Phillips, Glover Triplett, and Dave Van Dorn (Lessiter, 2018). No‐till systems developed by farmers throughout the Central US Great Plains were widely adopted in the 1990s because of their beneficial impacts on water relations, soil health, and farm productivity. These systems helped reverse summer‐fallow‐rainfall‐capture and alternate‐year cropping practices in the region initiating a “spiral of regeneration” where interactions among more favorable water relations, residue production, and crop yield were continually improving soil and landscape health and, consequently, future crop performance (Anderson, 2003).

Adoption of strip‐tillage surface biomass‐preserving practices in the southeast United States, also in the 1990s, was driven by the need to address hard‐setting compacted soils (Raper et al., 1994) and reduce production costs, all while being incentivized and required by the Food Security Act of 1985 to demonstrate cross‐compliance by having a conservation plan to reduce erosion on Highly Erodible Land when applying for government subsidies (Franklin & Bergtold, 2020). In the US Midwest states, Iowa, Illinois, and Ohio, maize/soybean/cover crop systems under no‐till are increasingly common today. Also, in the mid‐1990s, a few farmers from the semiarid northwestern US states, Washington, Oregon, and Idaho, visited Dwayne Beck at the South Dakota State University Dakota Lakes Research Farm in Pierre, SD, and the no‐till farmers he had been working with, to learn about the high residue no‐till systems they had been developing (Ross, 2005). These western areas average only 12–17 in. of largely winter precipitation each year when fields are typically bare and vulnerable to wind and water erosion. Realizing that a monoculture of continuous wheat was not sustainable without government subsidies, these farmers then returned home to convert their traditional dryland monocrop wheat and summer fallow rotation to a more diversified direct‐seeded no‐till annual cropping system that included wheat, safflower, sunflower, canola, and mustard.

**Core Ideas**

- California's Conservation Agriculture Systems Innovation (CASI) Center has fostered farmer‐led innovation for 25 years.
- Many education methods and >2000 participants (farmers, advisors, and researchers) are involved.
- Cover crops and no‐till increased soil quality and maintained crop productivity in a long‐term research trial.
- Conservation agriculture can potentially help California agriculture to remain viable under future water shortages.
- A group of >100 farmers and affiliates is exploring ways to develop organic conservation agriculture for California.

In each of these diverse regions, revolutionary change in the dominant cultures of agriculture that had prevailed began to take place in the late 1990s. Typical factors leading to these revolutions in all areas were (1) recognition of clear threats to farm profitability, such as soil erosion or the need to conserve soil moisture, (2) the need for systems thinking and problem‐solving rather than piecemeal, single remedies or adjustments, and (3) the creation of local networks of innovative farmers, professional advisors, and technologies that eventually developed into global knowledge‐sharing networks (Coughenor & Chamala, 2000; Kuhn, 1962). The history of revolutionary transformation toward CA in all of these regions was built by the many farmer‐led no‐till associations that were established in South America, in the Northwest United States by the Pacific Northwest Direct Seed Association (PNDSA) (https://www.directseed.org/), in the Great Plains United States and Canada by Manitoba North Dakota Zero‐till Farmers Association (https://www.no‐tillfarmer.com/keywords/16591‐the‐manitoba‐north‐dakota‐zero‐tillage‐farmers‐association), in the US Midwest by No‐till on the Plains (NTOP) (https://www.notill.org/) and the National No‐till Farmer (NNTF) (https://www.no‐tillfarmer.com/nntc), and in the southeast United States by the Southern Conservation Tillage Systems Conference (and subsequent Southeast Cover Crop Council), comprised of state groups such as the Georgia Conservation Tillage Alliance (www.gcta‐ga.org). The Conservation Agriculture Systems Alliance (https://www.ctic.org/resource_display/%22 http://www.ctic.org/Conservation%20Agriculture%20Systems%20Alliance/%22) was created in December 2007 by a consortium of these groups at a meeting in Pine Mountain, Georgia, to further build farmer‐led networks across North and South America. Until recently, these necessary elements for such wholesale revolutionary transformations in agricultural production systems have largely been lacking in California (Mitchell et al., 2024).

Adherence to CA principles is now widely known to result in many positive productivity, economic, environmental, and social outcomes (Kassam et al., 2020) including increased water infiltration and storage (Franzlubbers, 2010), decreased soil erosion (Ranaivoson et al., 2017) and soil water evaporation (Klocke et al., 2009), optimized soil moisture utilization (Nielsen et al., 2005) and nutrient cycling (Franzlubbers, 2010), and increased soil carbon stocks (Liptzin et al., 2022). In short, applying these principles regenerates a soil's productive capacity while performing vital ecosystem services (Mitchell et al., 2024), explaining why they have fueled a farming renaissance in several regions of the world (Anderson, 2005; Anderson, 2011; Crabtree, 2010; Kassam et al., 2022; Lindwall & Sonntag, 2010; Peiretti & Dumanski, 2014).

The modern version of CA is normally described (Kassam et al., 2022) as an ecosystem approach to regenerative, sustainable agriculture and land management, based on the practical application of the context‐specific and locally adapted three interlinked principles (limited or no mechanical soil disturbance, soil covered by biomass, and crop diversification) along with complementary practices including those related to integrated crop, soil, nutrient, water, pest, and energy management.

CA systems are present in all continents, involving rainfed and irrigated systems including annual cropland systems, perennial systems, orchards and plantation systems, agroforestry systems, crop‐livestock systems pasture and rangeland systems, organic production systems, and rice‐based systems. Conservation tillage (CT), reduced tillage, and minimum tillage are not CA, nor is no‐till on its own. A practice such as no‐till can only be referred to as being a CA practice if it is part of an actual CA system as per the above definition. This is similarly true for the soil mulch and crop diversification practices, both of which can only be considered to be CA practices if they are part of a CA system based on the application of the three interlinked principles.

Farmer‐led and farmer‐mentored networks developed rapidly throughout Brazil, Argentina, Paraguay, and Uruguay where crop subsidies such as those provided by the US Farm Bill do not exist. In Brazil, however, bank loans to farmers often require work plans showing CA adoption, indicating that the selection of farming practices is not entirely based on market‐driven decisions. Farmer‐led networks formed in the northern Great Plains of Alberta, Canada, where farmers travel great distances to share in farm tours, discussions, and visits with each other in a country with little government‐funded extension infrastructure. Farmer‐led networks developed rapidly in the US states Washington, Idaho, and Oregon through the now 30‐year‐old PNDSA to address soil erosion and water conservation goals; in the US southeast by the largely farmer‐led Georgia Conservation Tillage Alliance; and in proximity to the Dakota Lakes Research Farm in South Dakota to overcome the unsustainable economics of alternate‐year summer fallow practices.

In 2024, CA covered more than 260 million ha of annual global cropland (excluding pastures, orchards, and plantation crops), spread across more than 100 countries (T. Friedrich, personal communication, 2025), based on extrapolated estimates in Kassam et al. [2022], assuming the same growth rate used in that report). About 50% of the CA cropland area was located in the Global North and 50% in the Global South, benefiting millions of smallholders and larger‐scale farmers in all agroecological zones in all continents where agriculture is practiced. Since 2008/2009, the rate of adoption or implementation of CA cropland has been about 10 million ha annually. In addition, large areas of perennial CA systems, including orchards, vineyards, plantations, and annual cropping with trees, exist globally (Kassam et al., 2020; Kassam et al., 2022).

Despite the well‐documented agroecologically positive outcomes of employing CA principles, however, they have had only modest adoption in California, the leading agricultural state in the United States in terms of farmgate productivity and the crop species diversity of its agricultural output (CDFA, 2024). Indeed, the advent of well drilling and irrigation in the 1930s greatly expanded agriculture in California, along with improvements in crop genetics, more efficient farm equipment, improved cultural practices such as weed, disease, and insect control, as well as great strides forward in water management. Yields of all of the state's annual row and field crops that have been widely adapted to CA from other regions of the world have increased significantly since the start of recordkeeping in the late 1800s, leaving many wondering why farmers should change what they had been doing successfully for so long (CDFA, 2024). In the most current USDA National Agricultural Statistics Services Census of Agriculture for 2022, 3% of the cropland in California was under no‐till systems and 10% was under conservation or reduced tillage systems, defined as leaving “30 or more” or “15% and up to 30%,” respectively, of the soil surface covered by crop residue after planting based on total harvested cropland acreage (NASS, 2022). However, in the United States as a whole, no‐till CA systems occupied 38% of the annual cropland, and cover crops were planted on 4.7% of total US cropland in 2022.

Several factors explain the low adoption of CA in California's otherwise progressive and innovative agriculture systems. First, the historical motivators for CA adoption elsewhere—erosion control, water conservation, and reduced costs—have so far not been sufficiently strong to drive change toward CA in the State's major annual cropping systems. In 2002, our research team (see Section 2) conducted a survey of row crop producers in eleven Central Valley counties from Kern in the south to Yolo in the north to assess farmers’ familiarity with and general perceptions of CA (Mitchell, Klonsky et al., 2007). Major obstacles to broader adoption of CA by respondents included lack of information about CA, concerns that it would not work with certain crop rotations, and lack of interest in changing current practices. Later, in 2007, we identified other barriers to adoption: a lack of locally available CA equipment, inexperience with CA techniques, and the fact that existing tillage‐intensive systems have been productive for several decades (Mitchell et al., 2007). Even though we demonstrated that coupling no tillage with high residues could reduce evaporative losses by about 4 in., that is, 13%, assuming a seasonal crop evapotranspiration demand of 30 in. (Mitchell et al., 2012), California farmers did not adopt these practices, unlike farmers in other arid and semiarid regions. In sum, the State's unique crop diversity, unprecedented historical productivity, and the acquired familiarity with existing successful production systems have been prime reasons for why CA has not become more widely adopted in California.

Agricultural land with soil degradation and erosion as well as environmental pollution is, however, widespread in California. In addition, high costs of production in systems that are poorly adapted to climate change are concerns being increasingly voiced about California's existing and future productive capacity (ELJME, 2018). There is often the perception that little evidence exists in the historical literature that either wind or water erosion has been a major risk in California, in part because much of the cultivated land is on low slopes and precipitation occurs as low‐intensity storms (O'Geen et al., 2010), yet soil erosion detrimentally affects some 8.8 million acres (ELJME, 2018). Additionally, an estimated 419,000 tons (380,110 Mg) of nitrogen from nitrogen fertilizers leaches into California's groundwater annually, polluting it and contaminating drinking water (ELJME, 2018). Furthermore, because costs associated with tillage in annual crop production are typically less than 10% of total costs (Aegerter et al., 2023), economic drivers for reduced disturbance CA alternatives were not a priority for widespread adoption of alternatives to the dominant tillage‐intensive conventional systems of the past 90 years, especially as compared to improvements in crop genetics, pest management, and agronomy practices that have contributed to the large increases in yields of all major crops.

Context is critical for adoption of CA approaches to sustainable agriculture for both large‐ and small‐scale producers and for food systems that emphasize community‐based food production and consumption. CA recognizes the need to adapt farming practices to local landscape, climate, and cultural contexts, and support farming systems that are sustainable, productive, and also grounded in science (Mitchell et al., 2019). The objective of this review is thus to summarize the development and spread of CA systems in California and to recognize the diverse roles that various local and global farmer networks have had in its evolution.

## 2 CREATION OF THE CALIFORNIA CONSERVATION AGRICULTURE SYSTEMS INNOVATION CENTER

In 1998, the University of California's Division of Agriculture and Natural Resources (where the University's Cooperative Extension Service is administered) initiated a program aimed at forging stronger connections between campus‐based researchers and county‐based extension advisors through the creation of targeted workgroups to address emerging agricultural, environmental, and social issues in the state (https://ucanr.edu/sites/StrategicInitiatives/Program_Teams_Workgroups_Strategic_Initiatives/). The Conservation Tillage (CT) Workgroup coalesced at that time and was initially made up of a few University extension workers and researchers, pioneering farmers, several USDA Natural Resources Conservation Service (NRCS) conservationists, and private sector members. CT is defined by NRCS as any tillage and planting system that covers 30% or more of the soil surface with crop residue after planting to reduce soil erosion by water. This is now seen as a rather vague term that embodies nonspecific levels of soil disturbance (Reicosky, 2015). Tillage is a major cause of soil degradation and erosion. Beyond that early impetus to formally create the CT Workgroup, the University provided no further support. Also in 1998, a fax was sent out to local farmers in California's San Joaquin Valley (SJV) inviting them to learn about and develop information on CA practices and systems adapted to the region. At that time, the use of any sort of no‐ or minimum‐disturbance (no‐till or strip‐till) system was estimated to be extremely low, <2% of all cropped acreage in annual and field crop fields throughout the state (Mitchell et al., 2007).

The CT Workgroup eventually became the Conservation Agriculture Systems Innovation (CASI) Center in 2012 out of recognition that “conservation tillage” is truly an oxymoron (Reicosky, 2015) and that implementing the fundamental principles of CA involves more than just selecting new tillage equipment or conducting tillage system comparisons between conventional practices and slightly different mechanical alternatives (D. Beck, personal communication, 2018). Initially though, CASI emphasized opportunities through large‐scale equipment demonstration field days (some of which attracted over 300 participants) for people to network, learn about, and become familiar with the range of equipment options and approaches for various forms of reduced disturbance tillage. Local dealerships had little knowledge or familiarity with these implements especially because few were showcased at the popularly attended annual international farm equipment show held in Tulare, California.

During the late 1990s and early 2000s, CASI had a pivotal early role in introducing new pieces of specialized equipment, including no‐till planters, strip‐tillers, no‐till drills, high‐residue cultivators, no‐till transplanters, cover crop rollers, and choppers to farmers—all of which were acquired either through purchases through project grant support funding, donations, or equipment loan agreements with companies (totaling over $400,000). The CASI Center in Five Points, California, had a variety of transport trailers and flatbed trucks that allowed sharing of the equipment with farmers and demonstrating it at numerous public field days held throughout the Central Valley. These were inspired by the Jessup Agricultural Wagon of Dr. George Washington Carver of the Tuskegee Institute in 1906 (Tuskegee University, 2021) and later by the Research on Wheels program of Furney Todd of North Carolina State in 1975 (NCSU, 1975). As Mr. Todd was fond of doing in North Carolina, CASI often posted prominent signs along highly trafficked roads adjacent to CA demonstration fields.

**FIGURE 1:**
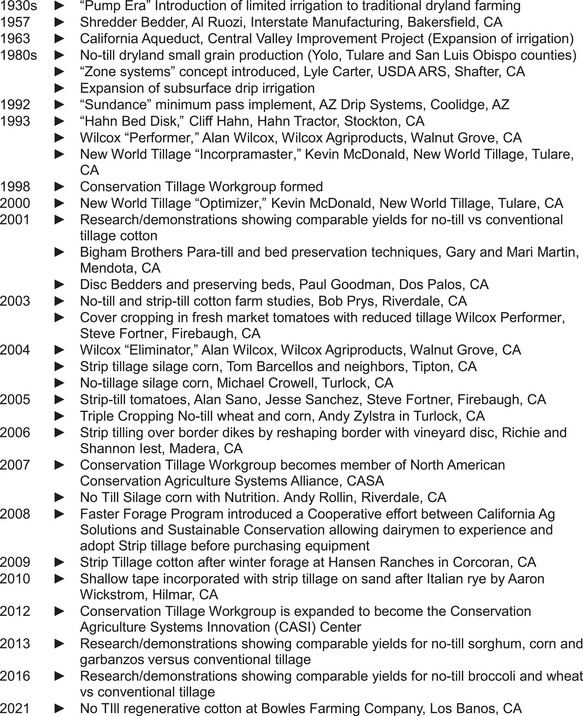
Advances in tillage management in California beginning with the advent of irrigation in the 1930s. This timeline chronicles significant introductions of technologies and use of reduced soil disturbance implements and approaches in California's annual cropping systems during this time.

A timeline of the history of advances in tillage management in California is shown in Figure 1. Particularly significant achievements include fabrication in 1957 by Bakersfield, California entrepreneur and CASI Center member, Al Ruozi, of a one‐pass “Shredder Bedder” machine that accomplished postharvest residue shredding with minimal soil disturbance and next‐crop planting bed preparation—a machine that appeared literally 50 years before other so‐called “minimum till” implements (https://ucanr.edu/blogs/blogcore/postdetail.cfm?postnum=15194). It combined the functions of tillage machines such as the “Hahn Bed Disk,” the “Wilcox Performer and Eliminator,” and the “New World Tillage Optimizer” that were introduced in the 1990s (Mitchell et al., 2016). Very early research on “zone tillage systems” by USDA ARS scientist and CASI‐recognized pioneer, Lyle Carter, eventually evolved and spread to strip‐tillage and other forms of vertical and precision tillage approaches that are common today (Carter et al., 1987; Mitchell, 2009) (https://ucanr.edu/sites/ct/Video_library/History_of_tillage_in_the_San_Joaquin_Valley/). Finally, a series of crop‐specific research evaluations tested the agronomic feasibility and development of new reduced disturbance production paradigms for cotton (Mitchell, Carter et al., 2012), tomatoes (Mitchell et al., 2012), sorghum (Mitchell et al., 2016), garbanzos (Mitchell et al., 2021) and wheat (Mitchell et al., 2021) in the Central Valley, a region that historically has relied on intensive disturbance during the past century. This agronomic work was accomplished in partnership with local SJV farmers who provided time, expertise, and field equipment (Mitchell et al., 2016), as well as with private sector CASI partners supplying equipment, guidance, e.g., for example, with on‐the‐ground experience with seedling establishment under high surface residue conditions, and education impact via their own farmer networks throughout California (Mitchell et al., 2012).

Because so much of the early work of the CT Workgroup and later CASI was new in California, its participating farmers and their allied private and public sector partners often initially worked in isolation and out‐of‐view of the mainstream. Eventually pockets, or “hotspots,” of coalesced progress by several farmers began to emerge. Thus, areas of the SJV around Tipton, Chowchilla, and Turlock first surfaced as places where concerted, and ultimately successful, work on CA dairy silage systems occurred. Similarly, the West Side SJV area around Firebaugh, California, was the initial site where a group of farmers pioneered reduced till and cover crop systems for processing tomatoes. Different California cropping systems emphasized CA principles that were most feasible to achieve or that farmers had tested. Thus, in almond and pistachio orchards, both single‐ and diverse‐species cover crop mixes started to be introduced around 2015 and onward (C. Crum, personal communication, 2022) where reduced tillage tended to already be the norm. Strip‐tillage and no‐tillage expanded in dairy silage production. Short‐season winter cover crops—both single species such as triticale (× *Triticosecale* Wittmack) and mixtures such as vetch (*Vicia sativa*), bell bean (*Vicia faba*), and winter peas (*Pisum sativum* subsp. *arvense*)—were often used in processing tomato fields with reduced‐pass tillage or strip‐tillage (Mitchell et al., 2009). During those early times, CASI played a useful and critical supporting role in terms of sharing information, equipment, and recently accumulated experience—both successes and failures—from other early CA adoption farmers who had taken on new and sometimes admittedly risky practices.

CASI was a true farmer‐based effort initiated by pioneering farmer leaders who were doing new things that had not been done before in California, like its predecessors, the PNDSA, NTOP, and the many South American networks mentioned above. Some recent so‐called “farmer‐led” initiatives are actually driven by university or government extension workers convincing farmers to trial a new practice on their land. The farmer networks that CASI emulated, and what it indeed created, were deliberate and true farmer initiatives. In much the same way as Indiana's Conservation Cropping Systems Initiative (https://www.ccsin.org/) charted a very aggressive strategic plan for accomplishing targeted numbers of annual extension education activities, CASI also was well‐served early on by a support and activity implementation plan that was developed by a small group of CASI university extension, NRCS, and ag‐support industry members over several months to give the Workgroup direction and benchmarks for achieving progress (https://casi.ucanr.edu/Mission_and_strategic_plan/). During its early evolution, CASI's development and expansion benefited greatly from direct visits and communications with several other similarly oriented, successful organizations such as the PNDSA, NTOP, and NNTF. Connections with these agencies and people helped CASI in event planning, hospitality, and program maintenance and fundraising. As visibility of CASI's efforts gradually increased during the early 2000s, it eventually began serving as a clearinghouse for information and help for farmer questions such as:

I've heard about folks doing strip‐till silage, but how can I get started with it?

I've got an NRCS EQIP contract for the “residue management” practice that my dad applied for, but now I don't know what to do.

In 2022, the market value of agricultural products sold by California's farms, ranches, and plant nurseries was $59.0 billion (CDFA, 2024). Not only is California diverse with over 400 crop commodities produced and sold annually, but the State leads the nation in the production of nearly 60 crops and is the sole producer (99% or more) of 12 important crops including almonds, celery, garlic, grapes, melons, pistachios, and walnuts. Early in the development of CASI, we made the decision to emphasize developing information for annual row and field crops of national or global importance (corn, cotton, wheat, beans, and cover crops). Tomatoes were added as an important crop in the Central Valley because of their requirement for minimum tillage due to widespread use of subsurface drip irrigation and also because relevant CA equipment was increasingly available, although not yet in California (Mitchell et al., 2007). Tillage intensity in many of California's permanent crops, such as almonds, pistachios, grapes, and oranges, already tended to be low relative to conventional tillage systems for annual crops. For example, data compiled by the California Almond Board's California Almond Stewardship Program in 2024 (https://almondstewardship.org) indicate that almond orchard floors are never tilled at 61% of California almond farms, tilled one to two times in the last 3 years at 24% of surveyed farms, and tilled only three or more times in the last 3 years at 5% of farms (G. Ludwig, personal communication, 2024). Orange groves are also typically untilled for the entire 40 or more years that they are productive.

## 3 GOALS OF THE CASI CENTER

In 2011, CASI began an open and transparent dialogue between about 30 volunteer members that was aimed at creating mission, values, and strategic plan statements to guide its activities. From this discussion, four goals for the workgroup were adopted in January 2012:

1. Develop and deliver information on the economic and environmental benefits of CA systems.
2. Increase understanding and adoption of locally appropriate CA systems to more than 50% of cropping acreage in California by 2028.
3. Partner with national and international conservation organizations and serve as a clearinghouse for information to promote CA systems.
4. Increase funding for CA systems research, education, and adoption in California.

Considerable internal discussion centered on whether the land‐grant university, as a critical player in CASI, ought to be behind actual adoption goals rather than the more neutral task of generating science‐based information. This subtle issue, plus the fact that CASI had very broad buy‐in from many diverse stakeholder groups including farmers, private companies, environmental groups, and other resource management organizations, set it apart from most other workgroups in the University of California system, and the adoption goal was kept (https://casi.ucanr.edu/Mission_and_strategic_plan/).

Funding support for CASI has been an ongoing challenge. The Workgroup has relied upon various public extension education programs such as state and national USDA NRCS Conservation Innovation Grants (CIGs) and the California Department of Food and Agriculture's Specialty Crop Block Grant Program for its organizational activities and a diverse portfolio of specific public and private sector grants to fund its applied research over the years. CASI never had high maintenance costs owing to its dedicated and committed core group of volunteers.

## 4 THE BEST EXTENSION EDUCATION PROGRAM IS MULTIPLE EXTENSION EDUCATION PROGRAMS

CASI's general approach has been that “the best extension education program is multiple extension education programs,” à la the many local and largely farmer‐led associations that have influenced its development and expansion. Its pursuit of multiple, diverse approaches—all aimed at increasing adoption—was born from recognizing two fundamental characteristics of CA. First, the “fundamentally new production paradigms” (in the words of CASI farmer member, Dino Giacomazzi of Hanford, California) that it sought to create and adopt in the Central Valley of California are not simple, single‐sorts of technology transfers or even more knowledge‐based management improvements such as integrated pest management or irrigation scheduling. Instead, they are more complex and systems‐based changes that required broad shifts of “mindset” through which farmers view agriculture systems and make farming decisions (Coughenour & Chamala, 2000). Wherever successful CA exists, the emphasis is on ecology‐based systems and how farmers develop an understanding of the changes they work to achieve. The second requirement for successfully constructing new cultures of agriculture based on principles of CA is the need, widely recognized worldwide, for local knowledge‐sharing networks of farmers, and often in partnership with the private sector (Coughenour & Chamala, 2000) as has been described above. The successful experiences of many of these global examples of local farmer‐led networks greatly informed the development and progress of CASI.

A significant aspect of the early and ongoing extension education work of CASI was the direct involvement and support of innovative farmers who began to learn individually about no‐till and strip‐till systems and who then volunteered willingly to share their knowledge with others in CASI‐sponsored activities. These farmers presented their progress to other farmers as well as to the more general public in farm field days, farm show presentations, documentary films, local press print outlets, and farm radio programs that are all archived at the CASI website (https://casi.ucanr.edu/). A tangible way in which the strength of CASI's diverse partners worked together is demonstrated by the action of a private sector member, Jerry Rossiter of CISCO AG, Atwater, California, who, on the evening before the original workgroup's formal public launching as the expanded CASI Center in 2012, hosted all workgroup members who were on the next day's speaking program in his motel room for live, stand‐up rehearsals of their presentations that afforded candid and valuable group feedback to everyone as a means to improve the quality of their speeches before they delivered them the following day to an audience of over 250 (https://ucanr.edu/blogs/blogcore/postdetail.cfm?postnum=8115). Out of the efforts to establish locally strong and lasting teamwork with CASI's diverse members, the center in 2017 further expanded its overall emphasis and goals to become a statewide farm demonstration network with hubs in several regions through a memorandum of understanding that was signed by the California Federation of Farm Bureaus, the California Department of Food and Agriculture, the California USDA NRCS, the California Association of Resource Conservation Districts, University of California Davis, and the University of California Division of Agriculture and Natural Resources (https://ucanr.edu/blogs/blogcore/postdetail.cfm?postnum=24054).

As with the numerous groups and networks that modeled success in diverse and multiple‐partnered CA organizations, farmers played pivotal roles in CASI. They actively contributed to the planning and hosting of educational events at their farms, at numerous large‐scale extension programs at CASI's SJV headquarters in Five Points, California (https://ucanr.edu/blogs/blogcore/postdetail.cfm?postnum=39614) and in numerous other public speaking sessions. They were accessible information sources for the CA systems that they had implemented and the equipment they had experimented with and answered phone calls from other farmers or offered actual in‐person help to farmers who were attempting new practices themselves. The extent of their networking generosity and open willingness to assist CASI and others who asked for their help was remarkable and very important to CASI's gaining momentum and broader visibility. Their support of on‐farm research led to numerous published articles on improvements in soil health, dust emissions, cover crop water use, fuel use comparisons, and the requisite “know‐how” for practice implementation.

Because so much of the experience base for CA systems in California did not exist, CASI has hosted US and international visitors who have shared their experiences with no‐tillage and CA (Table 1), typically at public events in Davis and Five Points, California, in the heart of the SJV. They were hosted at speaking events, farm visits and tours, and discussions with farmer groups and university students. Several of them have remained in ongoing connections with CASI and continue to provide ideas to discussions and educational programs.

**TABLE 1:** Some of the conservation agriculture experts who have been hosted by the CASI Workgroup to share their experiences with farmers in California.

Name	Location	CA intervention
Dick and Sharon Thompson	Iowa, USA	No‐till farmers and early mentors of Practical Farmers of Iowa
Dwayne Beck	South Dakota, USA	No‐till/regenerative agriculture researcher
Don Reicosky	Minnesota, USA	USDA ARS soil carbon CA scientist
Jerry Hatfield	Iowa, USA	USDA ARS research leader and CA scientist
Clay Mitchell	Iowa, USA	No‐till and strip‐till farmer innovator
Rolf Derpsch	Paraguay, South America	CA pioneer/educator
John McPhee	Tasmania, Australia	Government CA vegetable researcher
Ron Morse	Virginia, USA	University of Virginia cover and CA vegetable researcher
Max Carter	Georgia, USA	Strip‐till farmer and leader of the Georgia Conservation Tillage Alliance
Rick Reed	Georgia, USA	Coffee County, Georgia extension coordinator
Andy McGuire	Washington, USA	Extension soil scientist
Brendon Rocky	Colorado, USA	Colorado potato and cover crop farmer
Jay Fuhrer	North Dakota, USA	Retired USDA NRCS soil health conservationist
Li Hongwen	Beijing, China	Professor and CA leader
Steve Groff	Pennsylvania, USA	Farmer, cover crop and no‐till expert
Monte Bottens	Illinois, USA	No‐till corn, soybean, and cover crop farmer
Francis Akolbila	Ghana, Africa	Director of the Center for no‐till agriculture
John Luna	Oregon, USA	Extension educator and strip‐till vegetable researcher
Aref Abdul‐Baki	Maryland, USA	Retired USDA ARS cover crop research pioneer
Roberto Botelho Ferraz Branco	Brazil, South America	No‐till, cover crop research, and extension educator
John Landers	Brazil, South America	No‐till CA farmer pioneer and organizer

Abbreviations: CA, conservation agriculture; CASI, Conservation Agriculture Systems Innovation; NRCS, Natural Resources Conservation Service.

In 2005, CASI instituted an annual recognition program for CA farmers and private sector innovators in California. This acknowledgement effort became a well‐known program for the Workgroup that has identified and honored 22 true pioneers of CA in California (https://casi.ucanr.edu/) (Table 2). Criteria for these CASI Innovator recognitions are demonstrated innovation and leadership in the development, refinement, and use of CA systems within the California crop production environment. Nominations are carefully reviewed by a CASI Workgroup panel and recipients are announced in annual meetings. The major accomplishments of recipients are summarized in Table 2. Groups of CASI members have travelled to meet and learn from experts on various aspects of CA at the USDA ARS National Soil Dynamics Lab in Auburn, AL; the Georgia Conservation Tillage Alliance in Tifton, GA; the PNWDSA in Kennewick, WA; the USDA ARS National Lab for Agriculture and the Environment in Ames, IA; NNTF Conferences in St. Louis, MO; and Indianapolis, IN and the World Congress on CA in Winnipeg, Manitoba. In addition, in 2007, CASI took 12 California farmers to South Dakota, Nebraska, and Colorado to meet with no‐till farmers and researchers to learn about their systems. When members returned from these meetings, they had opportunities to report on and share what they had learned with other CASI members.

**TABLE 2:** Conservation agriculture farmer innovator award recipients 2005 through 2024.

Year	Farmer innovator	Town	Contribution
2005	Bob Prys	Riverdale, CA	No‐till, strip‐till cotton
2006	Tom Barcellos	Tipton, CA	No‐till, strip‐till dairy silage
2007	Jim Couto	Kerman, CA	One‐pass postharvest cotton residue/tillage equipment
	Tony Turkovich	Winters, CA	Cover crops and minimum tillage diverse crop rotations
2008	Dino Giacomazzi	Hanford, CA	Strip‐tillage dairy silage
2009	Alan Sano	Firebaugh, CA	Cover crops and strip‐till tomato
	Jesse Sanchez	Firebaugh, CA	Cover crops and strip‐till tomato
2010	John Diener	Five Points, CA	Strip‐till and cover crops for corn
2011	Steve Fortner	Brentwood, CA	Cover crops and strip‐till tomato
	Fred Leavitt	Exeter, CA	Cover crops and strip‐till tomato
	Fritz Durst	Dunnigan Hills, CA	No‐till dryland production
	Michael Crowell	Turlock, CA	No‐till dairy silage
2012	Gary and Mari Martin	Mendota, CA	Cover crops and minimum tillage
2013	Danny Ramos	Los Banos, CA	Cover crops and strip‐till tomato
	Ron Harben	Arroyo Grande, CA	Leadership contributions to CASI
	Ralph Cesena, Sr.	Stockton, CA	No‐till and ridge‐till pioneer
2015	Darrell and Trevor Cordova	Denair, CA	No‐till beans and small grains
	Charlie Rominger	Winters, CA	No‐till and cover crops diverse crops
2016	Mike Vereschagin	Glenn County, CA	Soil health management in orchards
	Mike Winemiller	Madera, CA	Conservation agriculture equipment and crop consultant
	Ladi Asgill	Petaluma, CA	Conservation Agriculture NGO consultant and organizer
	Steve Gruenwald	Orland, CA	Cover crops and strip‐till consultant
2017	Michael McRee	Chowchilla, CA	Strip‐till dairy silage
	Paul and Elizabeth Kaiser	Sebastopol, CA	Very small scale organic no‐till vegetables
2024	Scott, Ulla and Brian Park	Meridian, CA	Organic vegetables reduced disturbance tillage
	Paul Muller	Guinda, CA	Organic vegetables, no‐till, strip‐till
	Andrew Brait	Guinda, CA	Organic vegetables, no‐till, strip‐till
	Phil and Katherine Foster	Hollister, CA	Organic vegetables, cover crops, reduced disturbance tillage
	Tom Willey	Madera, CA	Organic vegetables, farmer organizer

Abbreviations: CASI, Conservation Agriculture Systems Innovation.

A final example of CASI's efforts to compile and share information about CA (initially, various forms of reduced soil disturbance tillage) in California are the surveys of annual crop acreage under different tillage management systems that were conducted in 2004, 2006, 2008, 2010, 2012, and 2014 based on estimates of over 35 local NRCS, University of California, and private sector experts (https://ucanr.edu/blogs/blogcore/postdetail.cfm?postnum=6429). Data were compiled for two general types of reduced disturbance systems. Practices such as no‐till, strip‐till, ridge‐till, and mulch‐till that leave at least 30% of the biomass residue from previous crops in place on the soil surface are the typical forms of reduced disturbance tillage in our survey. In addition, “minimum tillage” practices that reduce the overall number of tillage passes by at least 40% relative to what was done in the year 2000 were also included in the tally of tillage systems acreage.

In 2012, these “minimum tillage practices with much fewer tillage” (soil‐engaging) operations than in the year 2000 (Mitchell et al., 2015) accounted for about 17% of the total acreage for the crops that were surveyed, including silage and grain corn, small grains for hay, silage, and grain, tomatoes, cotton, dry beans, and melons throughout the nine‐county Central Valley region (https://ucanr.edu/blogs/blogcore/postdetail.cfm?postnum=15475).

The largest change in reduced disturbance tillage acreage from 2002 to 2012 was in the corn silage acreage that was strip‐tilled, a tillage system that only disturbs about a third of the total soil surface area of a field (Mitchell et al., 2015). In 2004, there were only about 490 acres of summer silage corn using strip‐tillage, while in 2012 over 181,000 acres (38.4%) throughout the SJV region had adopted the use of this form of reduced disturbance. Another trend during this time was the large increase in minimum tillage tomato acreage, rising from 3% in 2004 to about 58% in both 2010 and 2012. The corresponding increase in subsurface drip irrigation during this time, as mentioned above, precluded the ability to perform deep, broadcast conventional tillage and required use of shallower tillage that avoided damage to buried drip tape. Although the formal tillage management acreage survey stopped in 2014, minimum tillage in tomatoes has continued. However, there has been a drop‐off in the amount of new silage corn following strip‐till owing largely to a decline over time of personnel and capacity to try out new types of equipment and management practices within the CASI partners, as well as less technical service support by the private sector aimed at these areas (C. Crum, personal communication, 2024).

The outreach, extension, and farmer and industry education programs of CASI are widely recognized for their creativity and impact. The Center was an early adopter of using documentary film in science communication, and CASI's videos have had >1.2 million views on their YouTube channel (https://www.youtube.com/@jeffreymitchell759). Its work has reached wide popular as well as scientific audiences including a frequently accessed Grist.org essay (https://grist.org/food/no‐till‐farmings‐johnny‐appleseed‐in‐a‐grimy‐prius/), >250 web blogs at the CASI website (https://casi.ucanr.edu/), several installments of NRCS's Secrets of the Soil for California (https://www.nrcs.usda.gov/conservation‐basics/conservation‐by‐state/california/soil‐and‐soil‐health‐in‐california), hosting “Every Friday Soil Health Open House Tours” for 6 months at the 22‐year CASI study site in Five Points, CA, >600 public education demonstrations as part of CASI's annual presence at the World Ag Expo in Tulare, CA, and for about 1000 SJV 4th graders as part of their “Ag Ventures” educational program.

CASI received the Western Extension Director's Award for Excellence in Extension Education Programs in 2018, the National No‐till Farmers’ Organization Innovator Award in 2017, and the Group Innovator Award of the Soil and Water Conservation Society in 2022. CASI Chair, Jeff Mitchell, was recognized by the Research and Education Innovator Award of the National No‐till Farmer Association in 2020.

## 5 QUANTIFYING THE FEASIBILITY AND BENEFITS OF CONSERVATION AGRICULTURE IN CALIFORNIA'S CENTRAL VALLEY

**FIGURE 2:**
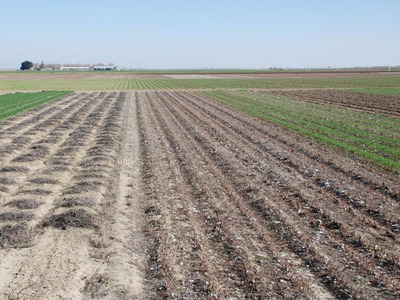
Overhead view of the 22‐year conservation agriculture systems research study in Five Points, CA. Begun in 1998, the study tracked changes in soil function indicators under conventional tillage with and without cover crops and no‐tillage with and without winter cover crops. No‐till no cover crop treatments in the middle of the photo(following tomato on the left and following cotton harvest on the right) with no‐till with cover crops (green swaths) just to the extremities of these.

The CASI Center has been a very early pioneer on several research fronts in California. An important initial and ongoing aspect of CASI's research is the evaluation of the impacts and tradeoffs that reduced disturbance tillage and cover cropping have on soil and cropping system function, water, carbon cycling, and economics. The Workgroup has led a long‐term cropping systems study to compare no‐till with winter cover crops (NTCC) and no‐till without winter cover crops (NTNO) to standard till with cover crops (STCC) and standard till without (STNO) cover crops (Figure 2) since 1999 in a research station trial at Five Points, California. The ability to generally maintain productivity using no‐tillage and cover crop practices has been shown for a variety of crops (Mitchell, Shrestha, Dahlberg et al., 2016 [sorghum]; Mitchell, Shrestha, Hollingsworth et al., 2016 [wheat]; Mitchell et al., 2015 [cotton], 2016 [corn], 2012 [tomato], 2021 [garbanzos]) despite the historical reliance on intensive soil disturbance. Early CASI research also showed that tillage costs were lower by an average of $70 per acre in 2011 (Mitchell et al., 2012) and fuel use was also reduced by about 12 gallons per acre. The long‐term trial showed that several important indicators of soil quality, including soil carbon and nitrogen, aggregation, and infiltration, result from these practices (Mitchell et al., 2015, 2017). Aggregate stability increased by at least twofold in the NTCC over the STNO treatment. Water infiltration rates were improved in the NT systems regardless of cover crop presence with a difference of almost two orders of magnitude between the least disturbed NTCC and the STNO systems. Soil carbon in the 0‐ to 15‐cm depth in the NTCC system (27.9 mt ha^−1^) was statistically higher than NTNO (19.6 mt ha^−1^) and STNO (19.1 mt ha^−1^), but not from STCC (23.0 mt ha^−1^) in 2019, 21 years since inception of the trial. Though tillage and cover crop management changed the distribution of carbon throughout the soil profile (0–90 cm), the systems were not statistically different (*p* ≤ 0.05) over the entire profile. Thus, this long‐term study shows significant positive changes in soil properties in the irrigated soils in the SJV due to the coupled impacts of no‐till and cover cropping practices. Recent projects report increases in key soil biological attributes including abundance and diversity of bacteria and fungi (Schmidt et al., 2019) and nematodes (Zhang et al., 2017). This research has generated a unique body of knowledge for the SJV, showing that reduced disturbance practices in this traditionally tillage‐intensive region decrease airborne dust (Baker et al., 2005; Madden et al., 2008, 2009) and greenhouse gas emissions (DeGryze et al., 2010) and provide annual cost savings of $50–$70 per acre. The study site in Five Points, CA, is the single most visited University of California research field in the state.

This CASI research study also showed that reducing tillage and maintaining crop residues on the soil surface may improve the water use efficiency of California crop production (Mitchell et al., 2012). In two field studies comparing no‐tillage with standard tillage operations (following wheat silage harvest and before corn seeding), we estimated that 0.89 and 0.97 in. more water was retained in the no‐tillage soil than in the tilled soil. In three field studies on residue coverage, we recorded that about 0.56, 0.58, and 0.43 inches more water was retained in residue‐covered soil than in bare soil following 6–7 days of overhead sprinkler irrigation. Assuming a seasonal crop evapotranspiration demand of 30 in., coupling no‐tillage with practices preserving high residues could reduce summer soil evaporative losses by about 4 in. (13%).

In addition to improving soil quality and cropping systems productivity, reducing dust production has important implications on air quality and human health, especially in California's SJV where agriculture is a significant contributor to particulate emissions. The adverse health effects of particulate pollution have been well‐described in the medical literature, with negative effects from both short‐ and long‐term exposure (Martinelli et al., 2013, US EPA, 2014). Chronic exposure to air pollution is associated with reduced life expectancy and numerous lung and cardiovascular diseases, including lung cancer, chronic obstructive pulmonary disease, asthma, ischemic heart disease, and high blood pressure (Health Effects Institute, 2020). Coccidiomycosis, also known as Valley Fever, named after the SJV, is an invasive fungal infection caused by inhaling dust containing *Coccidiodes* spores that are endemic to areas of the Southwestern United States including the SJV (Crum, 2022). Clinical manifestations are broad, with disease ranging from self‐limiting to more severe disseminated forms, which can involve multiple organ systems and may rarely be fatal (Zaheri et al., 2023).

The Workgroup's early research showed that particulate matter (PM) emissions resulting from routine conventional tillage operations can be reduced 75% or more using strip‐tillage and no‐till (Baker et al., 2005). In the early 2000s, eight of the counties in the central and southern SJV were in “serious” non‐attainment for PM10 emissions under the federal Clean Air Act (Ag Air Quality, 2004) and under the SJV Air Pollution Control District's Rule 4550, which required farmers to implement five Conservation Management Practices (CMPs) for each crop that they farmed to improve air quality. “Land preparation and cultivation” was one of the eligible categories for CMPs. Between 2000 and 2024, PM10 emissions in the SJV decreased 92.6 tons per day, of which 61.9 tons per day is attributed to agricultural sources that have CMPs per requirements in Rule 4550 (California Emissions Projection Analysis Model 2019 v1.03, annual average). PM2.5 emissions have decreased by 35.1 tons per day, of which 9.6 tons are attributed to agricultural sources subject to Rule 4550. The implementation of practices that result in less tillage disturbance are known to enhance human health and safety with tillage‐induced dust storms (Reicosky et al., 2023).

**FIGURE 3:**
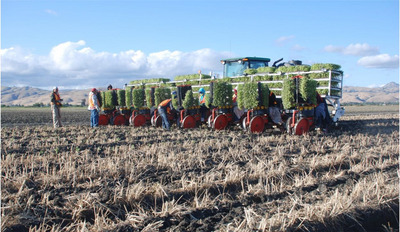
Large‐scale (eight 60‐in. rows) tomato transplanting into strip‐tilled cover crop beds, Hollister, CA, in field demonstration trial conducted by Conservation Agriculture Systems Innovation (CASI) Workgroup member, Danny Ramos, June 2010.

CASI has led an ongoing evaluation of tradeoffs between winter cover crop production and soil water depletion. In recent years, a growing number of SJV farmers recognize the value of using cover crops, but they are uncertain as to how these benefits are offset by the water use and cost of farming operations needed to grow a cover crop. Several local studies have now shown that soil water depletion under winter cover crops versus winter fallow is negligible in most water years (DeVincentis et al., 2022; Gomes et al., 2023; Mitchell et al., 2015, 1999). Another finding is that while vigorous growth of non‐irrigated winter cover crops in the SJV may not be possible in all years due to low and erratic precipitation patterns, the practice has benefits in many years. From 2022 to 2023, CASI partners provided 10 invited presentations to local Groundwater Sustainability Agencies, Resource Conservation Districts, and the California/Nevada Chapter of the Soil and Water Conservation Society about the long‐term benefits of cover cropping. This body of science has helped inform and shape policies related to California's Sustainable Groundwater Management Act in several SJV counties, and likely has contributed to the increases in cover cropping adoption in recent years in California that have now been documented (https://ucanr.edu/blogs/blogcore/postdetail.cfm?postnum=61550). It has been difficult to precisely track cover crop acreage in California due to fluctuating cash crop acreage and the fact that cover crops are not typically seeded over the entire soil surface in permanent crops as they are in annual crop fields. However, a January 2025 phone and email survey of the four largest cover crop seed companies in the state indicates an average annual increase in cover crop seed sales of about 23 ± 7% (mean ± SE) in recent years with a fivefold increase in overall cover‐cropped acreage since 2000. For example, one company reported an increase of 300,000 lbs of seed sold in 2024 compared to 2000, while another company's annual cover crop sales increased from about 10,000 lbs in 2016 to over 700,000 lbs in 2024. There are certainly multiple drivers responsible for these increases in adoption, including (1) incentive programs such as California's Healthy Soils Program (https://www.cdfa.ca.gov/oefi/healthysoils/), (2) the USDA NRCS EQIP Program, and (3) the 2021 adoption of the Ag. Order 4.0 regulation in the Central Coast region (https://www.waterboards.ca.gov/press_room/press_releases/2021/pr04162021_ag_order_4_0.pdf) that incentivizes cover cropping by providing cover crop nitrogen scavenging credits for nonlegume cover crops. The greater visibility of the concepts of regenerative agriculture in the state (https://www.cdfa.ca.gov/RegenerativeAg/) and CASI's research on cover crop water use and persistent extension education programs related to cover cropping have also likely had a role in these increased adoption trends (Figure 3).

CASI's research has also included a multi‐year USDA NRCS CIG involving organic farmers (plus >100 affiliates) investigating reduced disturbance practices and their impacts on soil function in organic vegetable crop systems. The project was driven by the practical interests and needs for information of these participating farmers and indeed was a rare example of farmer‐led research and education activity. Several types of reduced disturbance tillage, including strip‐tillage, in which only a band or strip of soil in the plant line is disturbed, a variant termed “vertical tillage,” in which flat disk blades penetrate the soil vertically to sever cover crop plant crowns and roots before vegetable transplanting, and a range of shallower‐depth tillage operations that performed full surface area tillage or soil disturbance, but to a shallower depth, were tried at each farm. No‐tillage was generally not attempted in these farm demonstrations owing to perceived difficulties and risks. Success was achieved for some crops with reduced disturbance approaches. By and large, however, reduced disturbance efforts failed to provide crop yields on a par with traditional organic tillage practices that had been developed and used for the past forty or so years. These organic farmers were not driven by yield alone and recognized the importance of a farm's long‐term viability, yet the magnitudes of lower productivity with the majority of CA approaches that they tried were not deemed sustainable.

Possible causes for the lack of success of the reduced disturbance organic systems include (1) lower nitrogen availability, particularly in shallow‐tilled soils with generally nonlegume cover crop mixes, (2) soil compaction, (3) seedling pests, (4) an inability to completely kill prior cover crops, (5) unincorporated cover crop residues that meant that their nitrogen is left on the soil surface or volatilized, becoming unavailable to vegetable cash crops, as well as (6) lower soil temperatures below these mulches compared to the customary clean‐bed surface conditions of previously developed organic tillage practices.

This project received national recognition as evidenced by an exhaustive case study published about project farmers (https://civileats.com/2021/03/30/can‐californias‐organic‐vegetable‐farmers‐unlock‐the‐secrets‐of‐no‐till‐farming/) and invitations to participating farmers to organize workshops at conferences of the Ecological Farming Association in Asilomar, California, in 2021, 2022, and 2024 (https://ucanr.edu/blogs/blogcore/postdetail.cfm?postnum=39294). The coupling of no‐till with organic vegetable production, however, proved so far to be risky and costly. Nonetheless, significant advances were made, such as shifts toward lighter and shallower disturbance, further expansion of the already considerable use of cover crops at these farms due to using reduced disturbance tillage approaches that require less time for incorporation, increases in the prevalence and duration of visible surface residues and living roots that persist throughout greater portions of the vegetable and cover crop rotations, and improvements in soil health that were of considerable importance to all participating farmers (https://ucanr.edu/blogs/blogcore/postdetail.cfm?postnum=50870).

CA is advancing in recent years in Central California, particularly in some cropping sectors, toward integrated, full‐system practices greatly enabled by progressive private sector consultants and support companies (https://tdwilleyfarms.com/feb‐5‐california‐ag‐solutions‐cary‐crum/). This private sector involvement with CASI has involved the active support from several CA equipment companies in sharing implements for CASI use, hosting jointly organized tours of CA demonstration fields and contributing to CASI strategic and specific event planning. CASI's company partners also have been critical in securing and fabricating state‐of‐the‐art cover crop seeding demonstration equipment, strip‐tillage equipment with precision fertilizer application capability, and farmer and farmworker CA equipment training workshops. Yet our results suggest that the vast majority of SJV annual crop farmers could greatly improve not only the function and efficiency, but also the sustainability, of their production systems by more widely adopting CA.

Despite its very earnest efforts, however, California's CASI Center will likely not reach its goal of 50% of the state's farming acreage under CA by 2028 based on the pace of current adoption trends. It has played an important role in introducing and focusing attention on production practices such as reduced tillage and cover crops that had not been part of the dominant farming paradigm of the past century. It coalesced a perhaps “once in a generation cohort” of diverse partners in ways that were modeled after the efforts of other similar leading groups of workers around the United States and world. This occurred during times when the funding support for such initiatives was not as available as it is today (https://www.cdfa.ca.gov/oefi/). Unlike in other regions where CA principles have been adopted, however, California has not yet confronted the combined economic and ecological drivers for large‐scale changes in production paradigms that have driven transformations that have occurred elsewhere where CA is now common. Informal surveys of the next generation of university, government agency, and even private sector contributors to the general area of work that CASI has pursued—soil health management—indicate that they are not likely to emphasize the admittedly “harder” aspects of it that include reduced disturbance tillage and surface residue preservation approaches due to the perceived inherent difficulties of successfully implementing these practices in California's high value, high risk annual crop environment. Although CASI's direct emphasis on these new (for California) reduced soil disturbance systems has now waned due to retirements and loss of new workers stepping forward to take up these challenges, other spin‐off groups in the state such as the University of California Extension‐led California Farm Demonstration Network (https://www.calfarmdemo.org/about‐cfdn/) and the private sector company, California Ag Solutions, that was founded in California by an Illinois no‐till farmer, Monte Bottens, who is a CASI member with considerable CA experience are now continuing CASI's early work.

## 6 THE GIANTS WHO CAME BEFORE AND WHO HAVE GREATLY HELPED CASI

At one of the luncheons of the annual meetings of the Southern Conservation Tillage Systems Conference (an association of groups throughout the southeast United States that worked to develop CA systems in their respective regions), American Society of Agronomy Fellow, National No‐till Farmer Association Legend, and former Ohio State University and then University of Mississippi soil agronomist, Glover Triplett, stood up and declared,

You know why I like to come to these meetings – it is because everyone here is working on hard problems.

In many ways, Triplett's sentiment aptly captures the challenges, motivations, and dedication that have influenced CASI—through a diverse array of connections—and its work in California. Interactions with the very early no‐tillers and partners like Glover Triplett in the US Midwest, as well as lessons from the numerous farmer‐led associations in Brazil, Argentina, Chile, and Paraguay, and the local organizations in the Pacific Northwest, the United States and Canadian Great Plains, and the US Deep South have helped CASI “work on hard problems” over the past 25 years. The benefits and sharing of ideas and information from these groups and the ability to readily interact with them have been enormous and greatly valued. Glover Triplett's “hard work” inspiration is important as we move forward to further evolution of CA systems into the future (D. Beck, personal communication, 2020).

Broad‐scale adoption of CA across more of California's diverse annual crop landscape is not going to be easy, yet the experiences and education of the last 25 years have been important and beneficial. The mechanical and biological techniques needed for the multitude of crops in typical rotations are just not available and have to date, not been shown to be reliable for all situations in which they are needed. Aspects of some of the work that CASI partners have started will continue in some fashion as part of the University of California's new emphasis on regenerative agriculture, for which new positions are being appointed and scoring platforms are being supported. One example is REGENScore (https://regenscore.org/), led by Jessica Chiartas with support from many CASI members, that uses “evidence‐backed, place‐based, and market‐driven” scoring frameworks and supply‐chain mechanisms to help farmers to transition to regenerative agriculture systems that are based on CA.

Lastly, Garrison Sposito, Distinguished Professor Emeritus at the University of California, Berkeley, and CASI member, has suggested that it may just be once or twice in a century that agriculture has an opportunity to re‐create itself in a revolutionary way (Johnson, 2014). For the many local farmer CA groups that have so graciously helped our CASI Workgroup by sharing their experiences and ideas over the years, that time has been during the past three or four decades. For California, there is optimism that it will hopefully be now.

Looking ahead, the adoption of CA systems in California will certainly continue, as more knowledge and evidence about the benefits of locally adapted CA systems and innovative practices spread through farmer associations, education, and governmental support, not only for annual crops but also for perennial crop systems such as vineyards, orchards, and plantations, which have been shown globally to benefit from CA. Policy support to enable farmers and institutions to work together, as associations and networks locally, nationally, and globally, will accelerate the uptake of CA across the state. Global scientific and empirical evidence strongly backs the claim that CA offers a practical opportunity to farmers and to the agriculture sector to improve agricultural performance in terms of crop and farm productivity and profitability as well as delivering ecosystem services to society. CA has the potential to enable agriculture in California to retain its role as a strategic player in the state and national economies.

## AUTHOR CONTRIBUTIONS

**J. P. Mitchell**: Conceptualization; data curation; formal analysis; funding acquisition; investigation; methodology; project administration; resources; supervision; writing—original draft; writing—review and editing. **L. E. Jackson**: Conceptualization; formal analysis; methodology; writing—original draft; writing—review and editing. **D. C. Reicosky**: Conceptualization; investigation; methodology; writing—original draft. **A. Kassam**: Conceptualization; methodology; resources; writing—original draft. **A. Shrestha**: Conceptualization; investigation; methodology; resources; writing—original draft. **R. Harben**: Conceptualization; investigation; methodology. **E. M. Miyao**: Investigation; resources. **K. M. Scow**: Conceptualization; investigation; methodology; writing—original draft; writing—review and editing. **G. Sposito**: Conceptualization. **D. Beck**: Conceptualization; investigation; resources. **Theodor Friedrich**: Conceptualization; resources; writing—original draft. **A. S. Mitchell**: Writing—original draft. **R. Scmidt**: Conceptualization; investigation; methodology; writing—original draft. **S. Park**: Investigation; resources. **B. Park**: Investigation; resources. **P. Foster**: Investigation; resources. **P. Muller**: Investigation; resources. **A. Brait**: Investigation; resources. **T. Willey**: Investigation; resources. **M. Bottens**: Investigation; resources. **C. Crum**: Investigation; resources. **D. Giacomazzi**: Investigation; resources. **T. Barcellos**: Investigation; resources **M. V. Crowell**: Investigation; resources. **R. Roy**: Investigation; resources. **H. Ferris**: Conceptualization; investigation; resources; writing—review and editing. **J. L. Chiartas**: Conceptualization; investigation; writing—original draft. **E. Brennan**: Investigation; resources. **A. Gaudin**: Conceptualization; investigation; resources. **John Diener**: Investigation; resources. **Justin Diener**: Investigation; resources. **L. Asgill**: Conceptualization; investigation; resources. **E. A. Kueneman**: Conceptualization; resources; writing—original draft. **J. Fisher**: Investigation; resources. **M. Bartz**: Resources; writing—original draft. **Roberto Atilio Peiretti**: Resources; writing—original draft. **R. Derpsch**: Conceptualization; resources; writing—original draft. **J. Landers**: Conceptualization; resources. **B. J. Aegerter**: Conceptualization; investigation. **M. Leinfelder‐Miles**: Conceptualization; investigation. **S. E. Light**: Conceptualization; investigation; resources. **J. McPhee**: Resources. **R. B. Ferraz Branco**: Conceptualization; investigation; resources.
